# Association of Pretreatment Serum Albumin and Systemic Inflammatory Markers with Pathologic Response to Neoadjuvant Chemotherapy in Breast Cancer

**DOI:** 10.3390/jcm15124429

**Published:** 2026-06-08

**Authors:** Selçuk Cin, Merve Tokocin, Özgecan Gündoğar, Merve Cin, Ali Muhammedoğlu, Murat Yüce, Ahu Senem Demiröz

**Affiliations:** 1 Department of Pathology, Cerrahpasa Faculty of Medicine, Istanbul University-Cerrahpasa, Istanbul 34098, Türkiye; ademiroz@iuc.edu.tr; 2 Department of General Surgery, Bağcılar Training and Research Hospital, University of Health Sciences, Istanbul 34200, Türkiye; mervetokocin@gmail.com; 3 Department of Pathology, Gaziosmanpaşa Training and Research Hospital, University of Health Sciences, Istanbul 34255, Türkiye; ozgecankarahan@hotmail.com; 4 Department of Pathology, Istanbul Training and Research Hospital, University of Health Sciences, Istanbul 34080, Türkiye; merveates42@hotmail.com; 5 Department of Pathology, Bağcılar Training and Research Hospital, University of Health Sciences, Istanbul 34200, Türkiye; alimuhammedoglu@hotmail.com; 6 Biomedical Engineering and Imaging Institute, Icahn School of Medicine at Mount Sinai, New York, NY 10029, USA; murat.yuce@mssm.edu

**Keywords:** pathologic complete response, breast cancer, neoadjuvant chemotherapy, serum albumin, Miller–Payne grading

## Abstract

**Background:** Pathological complete response (pCR) to neoadjuvant chemotherapy (NACT) in breast cancer is influenced by multiple tumor- and host-related factors, and readily available pretreatment biomarkers of response are still limited. This study aimed to evaluate the association between pretreatment systemic inflammatory and nutritional parameters and pCR assessed by the Miller–Payne grading system, with a specific focus on the independent predictive value of pretreatment serum albumin compared with established inflammatory ratios. **Methods**: A total of 226 patients with breast carcinoma who received NACT between May 2017 and September 2023 were retrospectively evaluated. Pretreatment laboratory parameters—including neutrophil-to-lymphocyte ratio (NLR), platelet-to-lymphocyte ratio (PLR), C-reactive protein (CRP), serum albumin, and the CRP/albumin ratio (CAR)—were recorded. Pathological response was assessed using the Miller–Payne grading system by two breast pathologists blinded to laboratory data. Univariable and multivariable logistic regression and receiver operating characteristic (ROC) curve analyses were performed, complemented by bootstrap validation of the optimal cut-off, a sensitivity analysis using the contemporary ypT0/is ypN0 definition of pCR, and a subgroup analysis by molecular subtype. **Results**: pCR was observed in 41 patients (18.1%). Pretreatment serum albumin levels were significantly lower in responders than in non-responders (*p* = 0.027), whereas NLR, PLR, CRP, and CAR were not significantly associated with response. In multivariable analysis, pretreatment serum albumin, Ki-67, and HER2 status emerged as independent predictors of pCR. ROC analysis demonstrated moderate discriminatory ability for albumin (AUC = 0.64); the optimal cut-off was 4.22 g/dL (bootstrap 95% CI 3.50–4.53 g/dL), with values below this threshold associated with a higher likelihood of pCR. The association between low pretreatment albumin and pCR was particularly pronounced in the triple-negative subgroup (3.30 vs. 4.02 g/dL, *p* = 0.027). The albumin signal remained significant under the stricter ypT0/is ypN0 definition of pCR in univariable analysis (OR 0.47, *p* = 0.045). **Conclusions**: Pretreatment serum albumin, independent of systemic inflammatory ratios, is associated with pCR to NACT in breast cancer and may serve as a candidate biomarker for pretreatment risk stratification, particularly when interpreted alongside established tumor-related predictors such as Ki-67 and HER2 status. The association appears especially relevant in the triple-negative subgroup, suggesting that patients with TNBC and low pretreatment serum albumin may warrant heightened multidisciplinary attention during NACT. Validation in larger, prospective, multicenter cohorts is needed before routine clinical implementation.

## 1. Introduction

Breast carcinoma represents the most frequently diagnosed malignancy in women and remains a major global health burden [1]. According to SEER cancer statistics, 316,950 new cases of breast carcinoma were diagnosed in 2025, with a substantial proportion presenting at a locally advanced stage [2].

Breast cancer is one of the best-characterized tumors at the molecular level, and complete treatment responses can now be achieved in certain patient subgroups [3]. Alongside ongoing molecular research, simple and widely available laboratory parameters—such as complete blood count (CBC) indices and biochemical markers—continue to attract attention as candidate predictors of prognosis and treatment response [1,4]. Tumor-infiltrating lymphocytes (TILs) have been extensively studied for their prognostic value across different solid tumor types. In breast carcinoma, particularly in HER2-positive and triple-negative subgroups, high TIL counts have been associated with a more favorable prognosis [5,6]. In contrast, increased neutrophil infiltration within tumor tissue is associated with adverse prognostic outcomes [4]. This effect is thought to be driven by mechanisms that facilitate tumor progression, such as enhanced angiogenesis and inhibition of apoptosis [7].

C-reactive protein (CRP) is an acute-phase reactant synthesized in the liver, and elevated levels have been associated with poor prognosis in breast cancer [8]. In contrast, albumin is a negative acute-phase reactant whose serum level also reflects systemic nutritional status. A growing body of evidence suggests that the CRP-to-albumin ratio (CAR) may serve as a prognostic indicator in breast carcinoma [8]. Notably, Hu et al. demonstrated that elevated CRP levels were associated with a complete response in hormone receptor–positive patients [9]. Collectively, these findings suggest that CRP and CAR may serve as biomarkers for risk stratification and for the assessment of treatment response in breast cancer.

Several classification systems are available for the assessment of pathological response to neoadjuvant therapy in breast cancer, of which the Miller–Payne grading system, developed in 2003, is one of the most widely applied. The scale ranges from 1 to 5; Grade 1 indicates no response to therapy or no significant reduction in tumor cellularity, and Grade 5 indicates a complete response with no evidence of invasive carcinoma [10]. Cases with no evidence of invasive carcinoma but with residual in situ disease are also classified as Miller–Payne Grade 5. Tumor response to therapy is determined by multiple factors, including histological type, tumor size, immunohistochemical and molecular features, and patient-related variables such as age, treatment regimen, and treatment compliance.

In this study, we aimed to evaluate the associations between the Miller–Payne treatment response and systemic inflammatory and nutritional markers—including neutrophil-to-lymphocyte ratio (NLR), platelet-to-lymphocyte ratio (PLR), CRP, albumin, and the CRP/albumin ratio—in patients with breast carcinoma treated with neoadjuvant chemotherapy, with a specific focus on the independent predictive value of pretreatment serum albumin. Although previous studies have separately addressed the prognostic value of either serum albumin or systemic inflammatory ratios in breast cancer, head-to-head comparisons of these markers within a single cohort and evaluated specifically against the Miller–Payne grading system are limited. The present study addresses this gap by directly assessing whether pretreatment serum albumin retains independent predictive value when analyzed alongside NLR, PLR, CRP, and the CRP/albumin ratio in the same patient population, thereby clarifying their relative contribution to the prediction of pathological response.

## 2. Materials and Methods

This study was conducted in accordance with the principles of the Declaration of Helsinki. Ethics approval was granted by the Ethics Committee of the University of Health Sciences, Istanbul Bağcılar Training and Research Hospital (Date: 17 November 2023; Approval Number: 2023/11/02/068). Pretreatment radiologic tumor size could not be reliably retrieved for all patients owing to the retrospective design of the study and heterogeneity in radiologic reporting across referring institutions; therefore, pretreatment tumor size was not included as a covariate in the regression analyses. In patients with residual disease at surgery, pathological tumor size was recorded for descriptive purposes, whereas in patients achieving a complete response no measurable residual tumor was present. For the same reason, post-treatment histological grade could not be determined in patients with pathological complete response, as no residual invasive tumor was available for grading; histological grade was therefore not included as a covariate in the multivariable model. Molecular subtype, although clinically informative, is fully derived from the immunohistochemical variables (ER, PR, HER2, and Ki-67) that were already included in the multivariable model; the contribution of subtype was instead explored through a dedicated subgroup analysis (Section 3.4).

### 2.1. Study Design and Setting

This was a retrospective single-center cohort study conducted at the Department of Pathology, University of Health Sciences, Istanbul Bağcılar Training and Research Hospital. Consecutive patients with biopsy-proven invasive breast carcinoma who received neoadjuvant chemotherapy followed by definitive surgical resection between May 2017 and September 2023 were identified through the hospital pathology database.

### 2.2. Patient Selection and Eligibility Criteria

Inclusion criteria were: (i) age ≥ 18 years; (ii) histologically confirmed invasive breast carcinoma on tru-cut core needle biopsy; (iii) completion of neoadjuvant chemotherapy followed by surgical resection at our institution; and (iv) availability of pretreatment laboratory parameters in the electronic medical record.

Exclusion criteria were: (i) metastatic disease at presentation (stage IV); (ii) prior chemotherapy received for any other malignancy; (iii) documented acute or chronic infection or systemic inflammatory disease at the time of pretreatment laboratory assessment; (iv) clinically or biochemically apparent hepatic or renal failure, which could independently affect serum albumin or C-reactive protein levels; and (v) missing essential clinicopathological or laboratory data.

### 2.3. Data Collection and Variable Definitions

Clinical, laboratory, and pathological data were retrieved from the hospital electronic medical records and pathology archive. Pretreatment laboratory parameters—including complete blood count (neutrophil, lymphocyte, and platelet counts), C-reactive protein, and serum albumin—were recorded from the most recent measurement obtained within four weeks prior to the initiation of neoadjuvant chemotherapy. Derived ratios were calculated as follows: the neutrophil-to-lymphocyte ratio (NLR) was calculated as absolute neutrophil count divided by absolute lymphocyte count; the platelet-to-lymphocyte ratio (PLR) as absolute platelet count divided by absolute lymphocyte count; and the CRP/albumin ratio (CAR) as serum CRP divided by serum albumin.

### 2.4. Neoadjuvant Chemotherapy

Patients received standard neoadjuvant chemotherapy regimens (most commonly anthracycline–taxane-based protocols) according to international guidelines, with the specific regimen and number of cycles determined by the treating medical oncologist on a per-patient basis. HER2-positive patients additionally received trastuzumab-based regimens per standard of care. Surgical resection was performed following completion of neoadjuvant chemotherapy, in accordance with standard institutional practice. Because individual regimen-level data were not consistently captured in the retrospective database, this information was not analyzed as a covariate; this is acknowledged as a limitation.

### 2.5. Pathological Response Assessment

Pathological response to neoadjuvant chemotherapy was assessed on surgical resection specimens using the Miller–Payne grading system [10]. The Miller–Payne system grades response on a five-point scale based on the change in tumor cellularity between the pretreatment core biopsy and the post-treatment surgical specimen: Grade 1, no change or some alteration to individual malignant cells but no reduction in overall cellularity; Grade 2, a minor loss of tumor cells (up to 30% reduction); Grade 3, an estimated reduction in tumor cellularity between 30% and 90%; Grade 4, a marked reduction with greater than 90% loss of tumor cells; and Grade 5, no residual invasive carcinoma, although ductal carcinoma in situ may be present. For analytical purposes, Miller–Payne Grade 5 was classified as pathological complete response (responders), whereas Grades 1–4 were classified as non-responders. Each surgical specimen was independently evaluated by two experienced breast pathologists who were blinded to laboratory data; in cases of disagreement, consensus was reached by joint review. To complement the Miller–Payne-based definition, a sensitivity analysis was additionally performed using the contemporary ypT0/is ypN0 definition of pathological complete response, in which complete response was defined as the absence of residual invasive carcinoma in both the breast (Miller–Payne grade 5) and the axillary lymph nodes (ypN0). Axillary nodal status was determined from the surgical pathology reports based on the number of involved lymph nodes among all examined nodes.

### 2.6. Immunohistochemistry

Estrogen receptor (ER), progesterone receptor (PR), human epidermal growth factor receptor 2 (HER2), and Ki-67 status were assessed by immunohistochemistry on available tumor tissue using the routine diagnostic protocol of our pathology laboratory, in accordance with current ASCO/CAP guidelines. A representative paraffin block containing the largest amount of tumor was selected from each case, and 4-µm-thick sections were stained on an automated immunohistochemistry platform (Ventana BenchMark ULTRA, Roche Diagnostics, Tucson, AZ, USA) according to the manufacturer’s protocol, using primary antibodies against ER (clone SP1), PR (clone 1E2), HER2 (clone 4B5), and Ki-67 (clone 30-9) (all Roche/Ventana). ER and PR were considered positive when ≥1% of tumor cell nuclei showed staining. HER2 was scored as 0, 1+, 2+, or 3+ according to standard ASCO/CAP criteria; HER2 IHC scores of 0 and 1+ were classified as negative, 3+ as positive, and 2+ cases underwent reflex silver in situ hybridization (SISH) using the INFORM HER2 Dual ISH DNA Probe Cocktail (Roche) on the same automated platform to determine final HER2 status. The Ki-67 proliferation index was reported as the percentage of positively stained tumor cell nuclei and analyzed in three categories: <10%, 10–20%, and >20%. In patients with pathological complete response, immunohistochemical assessment was based on the available pretreatment core needle biopsy material, as no residual tumor was present in the surgical resection specimens; for a subset of complete responders in whom pretreatment biopsy material was not retrievable, immunohistochemical results were not available.

### 2.7. Statistical Analysis

Analyses were conducted using SPSS version 25.0 (IBM Corp., Armonk, NY, USA). Continuous variables were summarized as mean ± standard deviation (SD) or median (range), and categorical variables as frequencies and percentages. Group comparisons between responders and non-responders were performed using the Mann–Whitney U test for continuous variables and Fisher’s exact test for categorical variables. Univariable logistic regression analyses were used to evaluate associations between clinical, laboratory, and pathological parameters and treatment response. In addition to variables with *p* < 0.05 in the univariable analysis, ER status was also included in the multivariable logistic regression model given its established clinical relevance. Odds ratios (ORs) with 95% confidence intervals (CIs) were reported. Model discrimination was assessed using the area under the receiver operating characteristic curve (AUC). Statistical significance was defined as *p* < 0.05. Missing data were handled by available-case analysis: each analysis was performed on all patients with complete data for the relevant variables, without imputation. The number of patients included in each analysis is reported in the corresponding table or figure. To assess the robustness of the optimal albumin cut-off value, 95% confidence intervals around the Youden-index-derived threshold were obtained using bootstrap resampling (1000 iterations).

## 3. Results

### 3.1. Descriptive Findings

A total of 226 patients were included in the study. The baseline clinicopathological and laboratory characteristics of the cohort, stratified by pathological response, are detailed in Table 1. Complete blood count and biochemical parameters were available for all cases. Hormone receptor status, HER2 expression, and Ki-67 proliferation index were assessed on available tumor tissue (see Section 2).

The mean age of patients was 51 years (range 25–79), with 118 (52.2%) aged ≤ 50 years and 108 (47.8%) aged > 50 years. According to the Miller–Payne grading system, pathological complete response (grade 5) was achieved in 41 patients (18.1%), grade 4 in 37 (16.3%), grade 3 in 91 (40.2%), grade 2 in 42 (18.5%), and grade 1 (no histopathological response) in 15 patients (6.6%).

Mean values of laboratory parameters across the cohort were: neutrophil count 4.0 × 10^9^/L, lymphocyte count 1.8 × 10^9^/L, platelet count 289 × 10^9^/L, NLR 2.8, PLR 185, serum albumin 4.1 g/dL, CRP 7.5 mg/L, and CRP/albumin ratio 2.1. Ki-67 data were missing for 9 patients; among the remaining cases, the mean Ki-67 proliferation index was 30%, with 35.9% of patients classified as <10%, 20.7% as 10–20%, and 43.3% as >20%.

Hormone receptor status was available for 219 of 226 patients. Among these, 76% were ER-positive and 21% were ER-negative, while 62% were PR-positive and 35% were PR-negative; the remaining cases had missing results. HER2 immunohistochemistry results were also available for 219 patients, with the following distribution: score 0 in 26.1%, score 1+ in 31.4%, score 2+ in 19.0%, and score 3+ in 20.3% (the remainder lacked complete classification). After reflex silver in situ hybridization (SISH) of HER2 IHC 2+ cases, complete HER2 status data were available for 212 patients, of whom 25.4% were classified as HER2-positive and 72.6% as HER2-negative.

The mean age of responders was 50.9 ± 2.0 years (range 28–79), compared with 51.1 ± 0.9 years (range 25–78) in non-responders; this difference was not statistically significant (*p* = 0.799). The mean post-treatment tumor size in the non-responder group was 3.0 ± 0.2 cm (range 0.1–20); in responders, no measurable residual tumor was present following neoadjuvant chemotherapy.

### 3.2. Relationship Between Neoadjuvant Therapy Response and Clinical, Laboratory, and Pathological Parameters

Among laboratory parameters, neutrophil, lymphocyte, and platelet counts, NLR, and PLR did not differ significantly between the two groups. Pretreatment serum albumin levels were significantly lower in patients with complete pathological response (Miller–Payne grade 5; mean 3.96 ± 0.49 g/dL) than in those with Miller–Payne grades 1–4 (mean 4.14 ± 0.49 g/dL; *p* = 0.027). Mean CRP levels were 6.6 ± 0.4 mg/L (range 0.4–32) in patients with Miller–Payne grades 1–4 and 10.3 ± 2.0 mg/L (range 2–67) in those with complete pathological response (*p* = 0.319). The mean CRP/albumin ratio also did not differ significantly between the groups (1.6 ± 0.1 vs. 2.7 ± 0.6, *p* = 0.185).

The Ki-67 index was significantly higher in patients with Miller–Payne grade 5 than in those with Miller–Payne grades 1–4 (48.3 ± 4.4 vs. 24.4 ± 1.6; *p* < 0.001).

ER status was significantly associated with pathological response (*p* = 0.001): patients with ER-negative tumors achieved Miller–Payne grade 5 more frequently than those with ER-positive tumors (15/48 [31.2%] vs. 19/171 [11.1%]). PR status was not significantly associated with response (*p* = 0.081); among PR-negative tumors, 17/79 [21.5%] achieved Miller–Payne grade 5, compared with 17/140 [12.1%] among PR-positive tumors.

Final HER2 status assessed by SISH was significantly associated with pathological response (*p* < 0.001). Patients with SISH-positive tumors had a higher rate of Miller–Payne grade 5 response than those with SISH-negative tumors (19/58 [32.8%] vs. 13/154 [8.0%]).

Histological grade could not be analyzed as a predictor of pathological response, because grade can only be assigned to specimens with residual tumor; in patients with Miller–Payne grade 5 (complete response), no residual tumor was available for grading. 

### 3.3. Regression Analyses and ROC Findings

In univariable analysis, pretreatment serum albumin (*p* = 0.027), Ki-67 (*p* < 0.001), and HER2 status (*p* < 0.001) were significantly associated with treatment response; higher Ki-67, HER2 positivity, and lower pretreatment serum albumin levels were all associated with a higher likelihood of pathological complete response.

In multivariable analysis, pretreatment serum albumin (*p* = 0.015), the Ki-67 index (*p* < 0.001), and HER2 status (*p* = 0.022) emerged as independent predictors of pathological response, with higher Ki-67, HER2 positivity, and lower pretreatment serum albumin all independently associated with a higher likelihood of complete response. Pretreatment serum albumin remained a robust and independent predictor of pathological complete response (OR 0.30, 95% CI 0.19–0.50, *p* = 0.015), alongside Ki-67 and HER2 status (Table 2).

**Figure 1:**
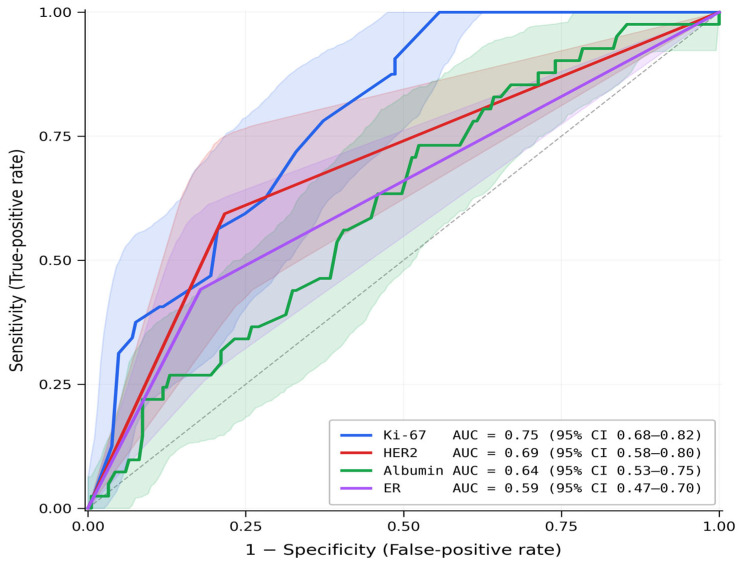
Receiver operating characteristic (ROC) curves for pretreatment serum albumin (AUC = 0.64, 95% CI: 0.53–0.75), estrogen receptor (ER) status (AUC = 0.59, 95% CI: 0.47–0.70), Ki-67 (AUC = 0.75, 95% CI: 0.68–0.82), and HER2 status (AUC = 0.69, 95% CI: 0.58–0.80) for predicting pathological complete response (Miller–Payne grade 5) to neoadjuvant chemotherapy. The *x*-axis represents 1 − specificity (false-positive rate) and the *y*-axis represents sensitivity (true-positive rate). The colored shaded areas represent the 95% confidence bands of the corresponding ROC curves, and the dashed diagonal line corresponds to no discrimination (AUC = 0.5). Among the variables tested, Ki-67 showed the highest discriminative performance, followed by HER2 and albumin.

ROC analysis showed that Ki-67 (AUC = 0.75, 95% CI: 0.68–0.82), HER2 (AUC = 0.69, 95% CI: 0.58–0.80), and albumin (AUC = 0.64, 95% CI: 0.53–0.75) had significant discriminatory ability for distinguishing patients with Miller–Payne grade 5 from those with Miller–Payne grades 1–4, whereas ER (AUC = 0.59, 95% CI: 0.47–0.70) showed weak discriminatory ability. The receiver operating characteristic (ROC) analysis of albumin, ER, Ki-67, and HER2 is presented in Figure 1. Multivariable logistic regression analysis identified Ki-67, HER2, and albumin as independent predictors of Miller–Payne response (model AUC = 0.84, *p* < 0.001). According to the Youden index, the optimal serum albumin cut-off value was 4.22 g/dL (bootstrap 95% CI: 3.50–4.53 g/dL; 1000 iterations). Patients with albumin levels below this threshold were significantly more likely to achieve a pathological complete response (Miller–Payne grade 5). At this cut-off, the sensitivity was 48% and the specificity was 73%. To assess whether the observed association was robust to the choice of pCR definition, a sensitivity analysis was performed using the contemporary ypT0/is ypN0 definition, in which complete response required absence of residual invasive carcinoma in both the breast and axillary lymph nodes. Of the 41 patients classified as responders by the Miller–Payne grade 5 criterion, 27 (65.9%) also met the ypT0/is ypN0 criterion, while the remaining 14 (34.1%) had residual nodal involvement. With this stricter definition, pretreatment serum albumin remained significantly associated with pathological complete response in univariable analysis (OR 0.47, 95% CI 0.22–0.98, *p* = 0.045). In the multivariable model adjusting for Ki-67, HER2 status, and ER status, albumin was no longer an independent predictor (OR 0.54, 95% CI 0.20–1.42, *p* = 0.211), most likely reflecting the reduced statistical power associated with the smaller number of events (*n* = 27) under the stricter definition.

**Figure 2:**
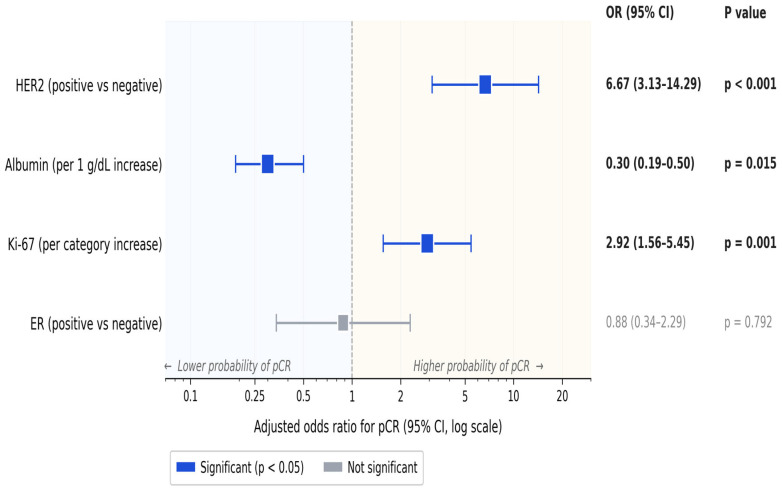
Forest plot of the multivariable logistic regression analysis for predictors of pathological complete response (pCR; Miller–Payne grade 5) to neoadjuvant chemotherapy. Squares represent adjusted odds ratios (ORs) and horizontal lines represent 95% confidence intervals. The vertical dashed line corresponds to OR = 1 (no effect). Estimates to the right of this line indicate a higher probability of pCR, whereas estimates to the left indicate a lower probability of pCR. HER2 positivity (OR 6.67, 95% CI 3.13–14.29; *p* < 0.001) and higher Ki-67 (OR 2.92, 95% CI 1.56–5.45 per category increase; *p* = 0.001) were independently associated with a higher probability of pCR, whereas higher pretreatment serum albumin (OR 0.30, 95% CI 0.19–0.50 per 1 g/dL increase; *p* = 0.015) was associated with a lower probability of pCR. Estrogen receptor status did not remain a significant predictor in the multivariable model.

Pretreatment serum albumin levels were additionally evaluated using an alternative dichotomization of Miller–Payne response (grades 4–5 versus 1–3); however, no statistically significant differences were observed. The adjusted effect sizes of the independent predictors of pathological complete response identified in the multivariable model are summarized graphically in Figure 2.

### 3.4. Subgroup Analysis by Molecular Subtype

To explore whether the association between pretreatment serum albumin and pathological complete response (pCR) differed across biological subtypes, patients were classified according to the surrogate molecular classification (Luminal A, Luminal B HER2−, Luminal B HER2+, HER2-enriched, and triple-negative). A total of 210 patients with complete data on ER, PR, HER2, and Ki-67 were classifiable; 16 patients (predominantly complete responders for whom no tumor tissue was available for full immunohistochemical evaluation) were excluded from this subgroup analysis. Pathological complete response rates varied markedly across subtypes, with the lowest rate in Luminal A (1.1%) and the highest rates in HER2-positive subtypes (Luminal B HER2+: 31.7%; HER2-enriched: 31.2%). In the triple-negative subtype, pretreatment serum albumin levels were significantly lower in responders than in non-responders (3.30 vs. 4.02 g/dL, *p* = 0.027), suggesting that the association observed in the overall cohort is particularly pronounced in this biologically aggressive subtype. In the other subtypes, no statistically significant association was identified; however, the limited sample sizes—particularly in the HER2-enriched group (*n* = 16)—reduce the statistical power of these comparisons. The results of the subgroup analysis are summarized in Table 3.

## 4. Discussion

In this study, we examined whether pretreatment neutrophil, lymphocyte, and platelet counts, together with NLR, PLR, CRP, serum albumin, and the CRP/albumin ratio (CAR), were associated with pathological response to neoadjuvant chemotherapy in patients with breast carcinoma.

The association between tumor-infiltrating lymphocytes and prognosis has been extensively investigated in numerous malignancies and continues to attract substantial scientific interest. Several studies have also examined complete blood count parameters as accessible and cost-effective biomarkers for predicting treatment response and prognosis. The role of neutrophils in cancer biology remains controversial. While neutrophils have been implicated in cytokine production and tumor angiogenesis, both in vivo and in vitro studies have also demonstrated their capacity to induce tumor cell death [4,11,12]. In numerous studies of breast cancer patients, elevated NLR and PLR have been associated with poor prognosis, axillary lymph node metastases, and unfavorable response to therapy [4,7,13,14]. In our cohort, however, no significant association was found between NLR and Miller–Payne response to neoadjuvant therapy. This finding contrasts with previous reports showing a significant association between elevated NLR and poorer therapeutic outcomes [14,15,16]. Consistent with our findings, Caziuc et al. reported no clear association between NLR and Miller–Payne response in a series of 96 patients treated with neoadjuvant chemotherapy [17]. Similarly, no statistically significant association was identified between PLR and Miller–Payne response in our cohort. The literature in this area remains heterogeneous, with no clear consensus established to date [14,17,18].

In this cohort, neither CRP nor CAR showed a significant association with Miller–Payne response to neoadjuvant therapy. This observation is consistent with the heterogeneous and frequently inconclusive results reported for pretreatment inflammatory and nutritional indices in breast cancer. A recent meta-analysis of pretreatment circulating inflammatory markers in the neoadjuvant setting concluded that the predictive value of these indices for pathological complete response is inconsistent across cohorts [19]. Similarly, a contemporary study using a comprehensive panel of inflammatory and nutritional indices (NLR, PLR, SII, PNI, HALP, LAR) reported conflicting predictive performance for individual indices [20]. More integrated composite measures—such as the neutrophil-to-albumin ratio—have been proposed to better capture both the inflammatory and the nutritional dimensions of the host response [21], suggesting that single-component inflammatory ratios may, in isolation, be insufficient. In contrast to these inflammatory and composite indices, pretreatment serum albumin—which serves both as a negative acute-phase reactant and as a marker of nutritional status—was significantly associated with treatment response when analyzed independently.

As a hepatically synthesized negative acute-phase reactant, albumin reflects both nutritional status and systemic inflammation, and reduced serum levels have consistently been associated with poor prognosis in patients with malignancies and in those undergoing major surgical procedures [22,23,24,25]. In patients with malignancy, the upregulation of proinflammatory mediators (e.g., IL-6 and TNF-α) suppresses hepatic albumin synthesis and contributes to the observed hypoalbuminemia [26]. Recent evidence in breast cancer treated with neoadjuvant chemotherapy has further refined the role of albumin-based predictors of pathological complete response. Zhang et al. constructed a nomogram incorporating the pretreatment serum albumin-to-alkaline phosphatase ratio (AAPR) together with histological grade and changes in tumor blood supply, demonstrating that AAPR was an independent predictor of pCR (OR 2.62, 95% CI 1.14–6.00) [27]. In a larger two-center cohort of 1170 patients, Qu et al. similarly developed and externally validated a prognostic nutritional index (PNI)-based nomogram, which combines serum albumin with peripheral lymphocyte count, and reported good discriminative performance for pCR prediction [28]. These findings are consistent with our results, which show that pretreatment serum albumin remains independently associated with pathological complete response when analyzed alongside Ki-67 and HER2 status, supporting the inclusion of albumin in composite predictive models. Earlier work, including a large prospective cohort by Tang et al. of approximately 4500 patients across multiple cancer types [22] and a report by Fujii et al. in 157 patients with breast cancer [29], had already linked low pretreatment serum albumin to adverse oncological outcomes; the present study extends these observations to pathological response prediction. Beyond breast cancer, comparable associations between low pretreatment serum albumin and adverse outcomes have been reported in colorectal [30] and gastric [31] malignancies, and serum albumin has also been shown to outperform prealbumin as a prognostic indicator in surgical oncology cohorts [32].

The biological associations between albumin and treatment response in malignancy are likely mediated by several complementary mechanisms, although these remain hypothesis-generating in the context of our retrospective observations. Albumin, through its Cys34 thiol residue, can scavenge reactive oxygen species generated during oxidative stress, which is implicated in tumor progression [33,34]. It also exerts immunomodulatory effects on B-lymphocyte and T-cell function [26,35], and, as the principal determinant of plasma oncotic pressure [36], it indirectly influences interstitial fluid pressure within the tumor microenvironment, an established barrier to drug delivery [37,38]. In addition, albumin functions as a major carrier protein that affects the transport and bioavailability of many therapeutic agents [39]. Taken together, these properties support interpreting albumin not solely as a nutritional marker but as an integrative parameter reflecting the inflammatory and immunometabolic state of the host, with potential consequences for chemotherapy response.

An additional finding of clinical relevance emerged from our subgroup analysis by molecular subtype (Table 3). Although the association between pretreatment serum albumin and pathological complete response was modest in the overall cohort, it was particularly pronounced in the triple-negative subgroup, where albumin levels were significantly lower in responders than in non-responders (3.30 vs. 4.02 g/dL, *p* = 0.027). Triple-negative breast cancer (TNBC) remains the most aggressive intrinsic subtype, characterized by the lack of targetable hormonal or HER2-driven pathways, and patients with TNBC continue to derive the greatest prognostic benefit from achieving pathological complete response after neoadjuvant chemotherapy [40]. The host inflammatory–nutritional axis, as captured by pretreatment serum albumin, may be particularly relevant in TNBC, where biological aggressiveness and systemic host factors interact closely. Although the limited number of TNBC patients in our cohort (*n* = 26) precludes definitive conclusions, this observation generates a testable hypothesis: that pretreatment serum albumin may carry greater discriminatory information in biologically aggressive subtypes than in luminal disease. Confirmation in larger, subtype-stratified cohorts is warranted.

Although our study did not identify significant associations between treatment response and inflammatory ratios such as NLR, PLR, or CAR, pretreatment serum albumin alone showed a significant and independent association with the Miller–Payne response. The discriminative ability of albumin was moderate (AUC = 0.64), with a cut-off value of 4.22 g/dL providing 48% sensitivity and 73% specificity. These findings suggest that pretreatment serum albumin may meaningfully contribute to pretreatment risk stratification when interpreted alongside established clinical and pathological parameters such as Ki-67 and HER2 status, rather than as a stand-alone marker. As a routinely available laboratory parameter, albumin therefore shows promise as a candidate biomarker.

From a clinical perspective, the present findings should be interpreted within a framework of supportive pretreatment risk stratification rather than as a stand-alone decision rule. Pretreatment serum albumin is a routinely available and inexpensive laboratory parameter, and a value below the optimal cut-off of 4.22 g/dL (bootstrap 95% CI 3.50–4.53 g/dL) was associated with a higher likelihood of pathological complete response in our cohort. However, the moderate discriminative ability of albumin (AUC = 0.64; sensitivity 48%, specificity 73%) indicates that this parameter should not be used in isolation to guide treatment decisions. Rather, the most appropriate clinical application is to interpret pretreatment serum albumin alongside established tumor-related predictors of response such as Ki-67, HER2 status, and hormone receptor status, which capture the underlying biological determinants of chemotherapy sensitivity. In the present study, the association between pretreatment serum albumin and pathological complete response was particularly evident in the triple-negative subgroup, suggesting that patients with triple-negative breast carcinoma and low pretreatment serum albumin may represent a subgroup in whom additional clinical attention—such as careful monitoring of nutritional status, supportive care, and consideration of multidisciplinary input prior to and during neoadjuvant therapy—may be especially warranted. Before pretreatment serum albumin can be formally incorporated into clinical decision algorithms, however, validation in larger, prospective, multicenter cohorts including diverse patient populations is required, ideally as part of composite models that integrate clinical, biological, and inflammatory parameters together with comprehensive assessments of treatment response. Recent work suggests that combining baseline and dynamic immune–nutritional indices during neoadjuvant chemotherapy may further enhance the predictive performance of these markers [41].

Several limitations should be acknowledged. Although a wide range of clinicopathological and laboratory parameters was evaluated, complete data could not be obtained for all variables. Nevertheless, the proportion of missing data was limited, and its impact on the overall results was considered minimal. In particular, pretreatment radiologic tumor size was not consistently available across the cohort owing to the retrospective design and heterogeneity in radiologic reporting; consequently, tumor size could not be included as a covariate in the multivariable model. Although tumor size is a recognized clinical predictor of response to neoadjuvant chemotherapy, the multivariable model incorporated other well-established predictors—including Ki-67, HER2 status, and hormone receptor status—that capture key biological determinants of chemotherapy sensitivity. Another limitation is that, in patients who achieved a complete response, receptor status (ER, PR, HER2, and Ki-67) could only be assessed on the pretreatment tru-cut biopsy specimens rather than on surgical resection material, because no residual tumor was present in the surgical specimens. Although hormone receptor status can shift following neoadjuvant treatment, the impact of this limitation on our findings is likely minimal. Finally, survival outcomes were not within the scope of this study and were therefore not analyzed.

The cohort of 226 patients was biologically heterogeneous with respect to hormone receptor status and molecular subtype, and the primary analysis was not pre-stratified by these subgroups. Future studies that pre-stratify patients by hormone receptor or HER2 status and establish subtype-specific serum albumin cut-off values may achieve greater discriminatory power for predicting Miller–Payne treatment response.

## 5. Conclusions

In this single-center retrospective cohort of 226 patients with breast carcinoma treated with neoadjuvant chemotherapy, pretreatment serum albumin—independent of systemic inflammatory ratios such as NLR, PLR, CRP, and CAR—was significantly and independently associated with pathological complete response, alongside Ki-67 and HER2 status. The discriminative ability of albumin was moderate (AUC = 0.64), with an optimal cut-off of 4.22 g/dL, indicating that this parameter should not be interpreted in isolation but rather alongside established tumor-related predictors of chemotherapy sensitivity. The association was particularly pronounced in the triple-negative subgroup, suggesting that patients with triple-negative breast cancer and low pretreatment serum albumin may warrant a heightened multidisciplinary approach—including nutritional assessment and supportive care—during neoadjuvant chemotherapy. As a routinely available and inexpensive laboratory parameter, pretreatment serum albumin shows promise as a candidate biomarker for pretreatment risk stratification; however, validation in larger, prospective, multicenter cohorts is required before routine clinical implementation, ideally within composite models that integrate clinical, biological, and inflammatory parameters.

## Figures and Tables

**Table 1:** Baseline characteristics by Miller–Payne response.

Variable	Category	MP 1–4 (*n* = 185)	MP 5 (*n* = 41)	*p*
Age (years)		51.1 ± 0.9 [25–78]	50.9 ± 2.0 [28–79]	0.799
Tumor size (cm) ^a^		3.0 ± 0.2 [0.1–20]	—	—
Neutrophil count (×10^9^/L)		4.1 ± 0.1 [1.0–10]	3.7 ± 0.2 [2.2–9.9]	0.123
Platelet count (×10^9^/L)		285.6 ± 5.6 [77–464]	299.9 ± 12.5 [125–457]	0.296
Lymphocyte count (×10^9^/L)		1.8 ± 0.1 [0.2–5.1]	1.9 ± 0.1 [0.7–3.7]	0.394
NLR		3.0 ± 0.3 [0.7–39.5]	2.3 ± 0.2 [0.7–10.3]	0.116
PLR		190.1 ± 10 [44–1168]	180.5 ± 15.4 [89–532]	0.597
Albumin (g/dL)		4.1 ± 0 [1.4–5.0]	4.0 ± 0.1 [2.7–5.1]	0.027
CRP (mg/L)		6.6 ± 0.4 [0.4–32]	10.3 ± 2 [2–67]	0.319
CRP/Albumin ratio		1.6 ± 0.1 [0.1–7.3]	2.7 ± 0.6 [0.5–21.4]	0.185
ER	Negative	33 (69%)	15 (31%)	0.001
	Positive	152 (89%)	19 (11%)	
PR	Negative	62 (78%)	17 (22%)	0.081
	Positive	123 (88%)	17 (12%)	
HER2	Negative	141 (92%)	13 (8%)	<0.001
	Positive	39 (67%)	19 (33%)	
Age category	≤50 y	96 (81%)	22 (19%)	0.864
	>50 y	89 (82%)	19 (18%)	
Ki-67	<10%	78 (100%)	0 (0%)	<0.001
	10–20%	38 (84%)	7 (16%)	
	>20%	69 (73%)	25 (27%)	

Continuous variables are presented as mean ± standard deviation [range], and categorical variables as number (percentage). Miller–Payne grades 1–4 were classified as non-response and grade 5 as complete response. NLR, neutrophil-to-lymphocyte ratio; PLR, platelet-to-lymphocyte ratio; CRP, C-reactive protein; ER, estrogen receptor; PR, progesterone receptor. ^a^ Tumor size after neoadjuvant chemotherapy was not measurable in MP 5 patients owing to complete regression.

**Table 2:** Univariable and multivariable logistic regression for predicting pathological complete response.

Variable	Univariable		Multivariable	
	OR [95% CI]	AUC [95% CI]	OR [95% CI]	*p*
Albumin	—	0.64 [0.53–0.75]	0.30 [0.19–0.50]	0.015
Ki-67	1.82 [1.55–2.13]	0.75 [0.68–0.82]	2.92 [1.56–5.45]	0.001
ER, Positive vs. Negative	0.27 [0.13–0.60]	0.59 [0.47–0.70]	0.88 [0.34–2.29]	0.792
PR, Positive vs. Negative	0.50 [0.24–1.05]	0.55 [0.43–0.66]	—	—
HER2, Positive vs. Negative	5.28 [2.40–11.64]	0.69 [0.58–0.80]	0.15 [0.07–0.32]	0.002
Age, >50 vs. ≤50 y	0.93 [0.47–1.83]	0.53 [0.42–0.65]	—	—

Multivariable analysis included albumin, Ki-67, HER2 status, and ER status. OR, odds ratio; CI, confidence interval; AUC, area under the curve. Dashes indicate variables not included in the multivariable model.

**Table 3:** pCR rates and pretreatment albumin levels by molecular subtype.

Molecular Subtype	*n*	pCR (*n*)	pCR Rate (%)	Albumin (g/dL): Resp vs. Non-Resp	*p*
Luminal A	94	1	1.1	3.98 vs. 4.20	0.439
Luminal B (HER2−)	33	7	21.2	4.19 vs. 4.10	0.809
Luminal B (HER2+)	41	13	31.7	4.01 vs. 4.20	0.274
HER2-enriched	16	5	31.2	3.72 vs. 3.84	0.212
Triple-negative	26	4	15.4	3.30 vs. 4.02	0.027

Continuous variables are presented as group means; *p* values were calculated using the Mann–Whitney U test. Subtypes were defined using surrogate criteria based on ER, PR, HER2, and Ki-67 status (Luminal A: HR+/HER2−/Ki-67 low; Luminal B HER2−: HR+/HER2−/Ki-67 high; Luminal B HER2+: HR+/HER2+; HER2-enriched: HR−/HER2+; Triple-negative: HR−/HER2−). Of 226 patients, 210 had complete immunohistochemical data and were classifiable.

## Data Availability

The datasets generated and analysed during the current study are not publicly available owing to patient privacy and ethical restrictions but are available from the corresponding author on reasonable request.
